# Pickering Emulsion Stabilized by Different Concentrations of Whey Protein–Cress Seed Gum Nanoparticles

**DOI:** 10.3390/foods13233777

**Published:** 2024-11-25

**Authors:** Maryam Davtalab, Sara Naji-Tabasi, Mostafa Shahidi-Noghabi, Artur J. Martins, Ana I. Bourbon, Miguel A. Cerqueira

**Affiliations:** 1 Department of Food Nanotechnology, Research Institute of Food Science and Technology (RIFST), Mashhad 91895-157-356, Iran; m.davtalab@rifst.ac.ir; 2 Department of Green Technologies in Food Production and Processing, Research Institute of Food Science and Technology (RIFST), Mashhad 91895-157-356, Iran; m.shahidi@rifst.ac.ir; 3 International Iberian Nanotechnology Laboratory, Av. Mestre José Veiga s/n, 4715-330 Braga, Portugal; artur.martins@inl.int (A.J.M.); ana.bourbon@inl.int (A.I.B.); miguel.cerqueira@inl.int (M.A.C.)

**Keywords:** *Lepidium sativum* L., nanomaterial, surface tension, emulsion

## Abstract

Nanoparticles based on food-grade materials are promising materials to develop Pickering emulsions for food applications. Initially, this study focuses on the development of nanoparticles through the utilization of a soluble complex of whey protein concentrate (WPC) and cress seed gum (CSG), which were modified by calcium chloride (CaCl_2_) as a cross-linker. The response surface methodology was used to investigate the impact of different concentrations of WPC (1–4% *w*/*v*), CSG (0–1% *w*/*v*), and CaCl_2_ (1–3 mM) on particle size, polydispersity index (PDI), and Zeta potential. The optimum conditions for the production of CSG–WPC nanoparticles (WPC–CSG NPs) were 0.31% (*w*/*v*) CSG, 1.75% (*w*/*v*) WPC, and 1.69 mM CaCl_2_, resulting in nanoparticles with average size of 236 nm and Zeta potential of −22 mV. Subsequently, oil-in-water (O/W) Pickering emulsions were produced with different concentrations of WPC–CSG NPs in optimum conditions. The contact angles of the WPC–CSG NPs were 41.44° and 61.13° at concentrations of 0.5% and 1%, respectively, showing that NPs are suitable for stabilizing O/W Pickering emulsions. Pickering emulsion viscosity rose from 80 to 500 mPa when nanoparticle concentration increased from 0.5% to 1%. Results also showed that WPC–CSG NPs enable stable O/W Pickering emulsions during storage and thermal treatment, confirming that protein–polysaccharide NPs can provide a sufficient steric hindrance.

## 1. Introduction

Pickering emulsion is popular in pharmaceutical, cosmetic, and food fields due to its stabilization by non-toxic, eco-friendly solid particles, and high stability [1]. Pickering emulsions, stabilized by solid particles, offer enhanced stability compared to traditional surfactant-based emulsions. Classic emulsions, stabilized by molecular surfactants, often exhibit limited long-term stability and can pose environmental risks. Unlike surfactants, solid particles directly interact with the oil–water interface, improving stability. The wettability of these particles determines the emulsion type (oil-in-water or water-in-oil) [2]. High internal phase emulsions (HIPEs) are characterized by having a dispersed phase volume greater than 74%. HIPEs, which can convert liquid oil into solid oil without harmful trans fats, are a promising application. Nanoparticles, with their adjustable polarity, are ideal for stabilizing Pickering emulsions, addressing consumer health concerns related with the use of synthetic surfactants [3].

The food-grade particles that stabilize Pickering emulsions can be based on polyphenols, proteins, and polysaccharides. Although proteins are known for their surface activity and rapid adsorption kinetics, some phenomena, such as aggregation and structural expansion, can reduce their emulsifying ability [4,5]. Polysaccharides are typically hydrophilic molecules, which makes them less effective at lowering the interfacial tension between oil and water, which is a key property of good emulsifiers [6].

On the other hand, Pickering emulsions stabilized by proteins–polysaccharides had increased stability and absorption of the active substance proficiency than the ones stabilized by proteins [7,8]. It has been reported that the protein–polysaccharide complex increases the steric layer and helps the electrostatic repulsion between droplets, increasing emulsion stability [7,9]. In addition to their crucial role in stabilizing Pickering emulsions, the wettability of nanoparticles can be enhanced by utilizing the hydrophilic properties of polysaccharides to improve the water solubility of proteins, thereby increasing the wettability of particle complexes [10,11]. This allows the particle to sit at the interface, forming the mechanical barrier between the two phases [12,13]. The wettability of nanoparticles is enhanced through their interaction with proteins and polysaccharides, enabling them to better distribute themselves at the boundary between oil and water [14,15].

Among biopolymers, whey protein is broadly used as an emulsifier, foaming, and gelling agent in the drug and food industry. Furthermore, the choice of protein and polysaccharide materials to form a suitable nanocomplex system depends on compatibility with each other to create a composite with special properties such as smaller particles’ size, more surface charge, or active functional groups. pH, ion, or concentration of polymer solution influences the nanoparticle properties [16]. The pH conditions exerted a noteworthy influence on the electrostatic interactions and thermal stability of the nanoparticle complex [17,18]. The electrostatic interaction and the hydrogen bond play an important role in protein–polysaccharide complexes [19]. The ionic strength can significantly affect nanoparticles from protein–polysaccharide complexes. It is known that calcium ions can create a salt bridge between proteins and polysaccharides and improve their stability [20]. The lower positive charge of protein exists above isoelectric points (*pI*), so the weak electrostatic interactions between proteins and polysaccharides create soluble complex [21]. The smaller sizes and higher absolute values of Zeta potential observed in soluble protein–polysaccharide complexes compared to insoluble complexes enhanced their functionality as nutrient delivery systems [22].

Cress seed gum (CSG) (*Lepidium sativum* L.) has steadiness in a wide range of temperatures, pH, and ion salt in solution. CSG is a galactomannan with a high mannose-to-galactose ratio (M/G of 8.2). The sugar profile is characterized by the presence of various sugars such as mannose (38.9%), arabinose (19.4%), galacturonic acid (8%), fructose (6.8%), glucuronic acid (6.7%), galactose (4.7%), rhamnose (1.9%), and glucose (1.0%) [23,24]. The sugar profile analysis of CSG revealed a higher ratio of D-galacturonic acid and D-glucuronic acid, which is characteristic of natural polyelectrolyte polysaccharides. These polyelectrolytic and anionic properties of CSG can facilitate the formation of protein–polysaccharide complexes through electrostatic interactions [25,26].

Considering the unique properties of CSG as a novel source of carbohydrates, so far, no research has been performed on its application and improving the functional properties of whey protein concentrate (WPC) for use in encapsulation and stabilization. Therefore, in this work, the optimal conditions for soluble complexes were determined by adjusting pH, salt (calcium chloride), and biopolymers ratio (WPC and CSG). The size distribution, PDI, Zeta potential, and morphology were used to optimize conditions for WPC–CSG NPs. Afterward, the properties of Pickering emulsion stabilized by whey protein complexes with CSG in different concentrations were investigated, and the adsorption mechanism in oil–water interfaces was explored.

## 2. Materials and Methods

### 2.1. Materials

CSG (*Lepidium sativum* L.) was purchased from a local market (Mashhad, Iran). Whey protein concentrate (WPC) (protein content: 85% (d.b)) was obtained from Hilmar Company (Hilmar, CA, USA). Calcium chloride (CaCl_2_) and other chemicals were supplied from Sigma-Aldrich Company (Steinheim, Germany). Fluorescent agents, Nile red (99%), were purchased from Sigma-Aldrich (Germany). Corn oil (Fula, Sovena, Portugal) was purchased from the local market (Braga, Portugal). All the chemicals used were of analytical grade.

### 2.2. Extraction of CSG

The extraction of CSG was conducted following the methodology established by Naji-Tabasi et al. (2016). Briefly, the cress seeds were cleaned, then submerged in deionized water (water–seed ratio of 37:1 at 40 ± 1 °C and pH = 7), and stirred for 40 min. The mucilage was scraped with a rotating abrasive plate. The extracted mucilage was poured into ethanol to purify. Also, the obtained stock solutions with different concentrations of CSG for more purification were centrifuged at 7100× *g* at 10 min to separate undesirable ingredients. Finally, the gum powder was obtained by drying in an oven (Memert-UF55: Schwabach, Germany) at 60 °C in airflow conditions [27].

### 2.3. Turbidity of WPC–CSG Complex Based on Shifts in pH Values

The WPC solution (1% *w/v*) was prepared and left overnight. Afterward, the solution was heated at 80 °C for 30 min and immediately cooled. CSG (0.25% *w*/*v*) and WPC (1% *w*/*v*) with a 50:50 ratio were mixed. The pH of the CSG–WPC mixtures was adjusted in the range of 3–7 by dropwise addition of HCl/NaOH (0.1 M) under stirring at a constant rate of 300 rpm. The pH was diligently observed using a benchtop pH meter (Metrohm-780, Herisau, Switzerland) throughout the process. Solutions were vortexed for 23 s before turbidity reading at room temperature (25 ± 2 °C). Dispersion absorption was measured at 500.0 nm with a UV-visible spectrophotometer (DR 5000, Hach, Germany) [28].

### 2.4. Preparation of WPC–CSG NPs

In the first step, WPC solutions (1–4% *w*/*w*) and CSG solutions (0–0.8% *w*/*w*) were prepared and both of them were kept overnight to complete hydration at 4 °C. WPC solution was heated at 80 °C for 30 min and immediately cooled. Then, the CSG and WPC solution were mixed (50:50) by using a magnetic stirrer with a speed of 400 rpm for 1 h. pH of dispersions was adjusted around 6.0 ± 0.1 to create a soluble complex.

For the preparation of the WPC–CSG complex, CaCl_2_ (1–2.6 mM) was added dropwise to dispersions under magnetic stirring (3000 rpm) at ambient temperature. The solution was then immediately sonicated by ultrasound (VCX750, SONICS Company, Hounslow, UK) at an amplitude of 80% with 5 s pulse on and 5 s pulse off, for 10 min in an ice bath for controlling temperature; 0.02% (*w*/*w*) sodium azide used to prevent bacterial growth [29].

### 2.5. Particle Size Distribution of WPC–CSG NPs

Dynamic light scattering (Malvern Company, Worcestershire, UK) was used for measuring WPC–CSG NPs (diluted with deionized water at a ratio of 1:10) hydrodynamic diameters and PDI at 650 nm. The diluted samples at a temperature of 25 °C are subjected to a glass cuvette with square aperture (No: ZEN1002). All measurements were repeated at least three times.

### 2.6. Zeta Potential of WPC–CSG NPs

The Zeta potential of WPC–CSG NPs was evaluated by a Zetasizer (Cordouan Technologies, Pessac, France). The samples were diluted with deionized water at a ratio of 1:10. The diluted sample was subjected to electrophoresis at 100 V in the Folded Capillary Zeta Cell, and the droplet surface charge was calculated from the droplet mobility at 25 °C and laser wavelength of 633 nm. All measurements were repeated at least three times.

### 2.7. Morphology of WPC–CSG NPs

The morphology of nanoparticles was observed by atomic force microscopy (ARA-AFM, Tehran, Iran). The samples were dried on a mica plate, and AFM images were acquired in tapping mode at a constant temperature (25 °C).

### 2.8. Water Contact Angle

The contact angle of optimized nanoparticles at different concentrations (0.5, 0.7, and 1%, *w*/*v*) was investigated on glass microscope slides. The glass slides were immersed in 100 mL fresh nanoparticle suspension (5 h), and excess polymer suspension was removed. The treated slides were dried in an oven at 30 °C for 24 h to create a thin film of attached nanoparticles. The five glass slides were prepared for each sample under this condition. The contact angle results are reported through the water phase, and water droplets were spotted on surfaces covered with nanoparticles via the micro-syringe. A drop of distilled water (2 μL) was dropped slowly on the surface and after equilibration, and the drop images were captured at room temperature [30] using a highspeed camera (9 megapixels, TSVIEW, Fouzhou, China). The drop images were fitted using the Younger–Laplace equation [31], the contact angle images were analyzed through Image J software (Image J 1.46 r, USA), and all measurements were carried out in triplicate.

### 2.9. Tensiometer

The WPC–CSG NPs and WPC NPs were prepared at optimum conditions according to previous results. The interfacial properties of nanoparticle-stabilized Pickering emulsion were measured by using an interfacial Tensiometer (K100, KRUSS Company, Hamburg, Germany).

### 2.10. Pickering Emulsion Properties

#### 2.10.1. Preparation of Pickering Emulsion

The Pickering emulsion samples were prepared by using WPC–CSG NPs at three concentrations of 0.5, 0.7, and 1% (*w*/*v*) as the continuous phase of Pickering emulsion. The corn oil fraction (15% (*v*/*v*)) was added to solid particle dispersions (85% *v*/*v*) according to the previous work [32]. The pre-emulsions were then homogenized using ultraturrax (T25 digital Ultraturrax, Staufen, Germany) at 17,000 rpm for 10 min. The resulting secondary particle-stabilized emulsions treatment by sonication (an amplitude of 45% with 3 s pulse on and 3 s pulse off, for 5 min). The stabilized emulsion samples were prepared in triplicates. Sodium azide (0.02% *w*/*v*) was added to the emulsions to prevent microbial growth during refrigerated storage at 4 °C.

#### 2.10.2. Droplet Size, PDI, and Zeta Potential

Zeta potential measurements of Pickering emulsions were conducted by Zeta Potential Analyzer (SZ-100, HORIBA Company, Kyoto, Japan) at 25 °C. The samples were diluted with deionized water at a ratio of 1:10,000. The diluted emulsion was subjected to electrophoresis at 100 V in the Folded Capillary Zeta cell, and the droplet surface charge was calculated from the droplet mobility at a laser wavelength of 633 nm. Also, the samples for droplet size were diluted with deionized water at a ratio of 1:1000 and subjected to 14 mm square polystyrene cuvettes (DTS0014) at 25 °C to determine the size and PDI. All measurements were repeated at least five times.

#### 2.10.3. Rheology

Rheological measurements of Pickering emulsions stabilized by different concentrations of WPC–CSG NPs (0.5, 0.7, and 1%, *w*/*v*) were performed by an MCR 302 rheometer (Anton Paar GmbH, Graz, Austria) equipped with a 50 mm cone-plate probe and set to a gap width of 0.096 mm. The flow behavior of Pickering emulsion was measured as the ratio of shear stress to shear rate (0–100 1/s).

#### 2.10.4. Confocal Laser Scanning Microscope (CLSM) Analysis

The morphology of Pickering emulsions stabilized by WPC–CSG NPs (0.5, 0.7, 1%, *w*/*v*) was performed with a confocal laser scanning microscope (Zeiss LSM 780 inverted, Jena, Germany). Before observation, Nile red (0.01% (*w*/*v*)) was used as a dye of the dispersed phase (oil). Then 100 µL of emulsion was mixed with 10 µL fluorescent dye. After staining, the Pickering emulsion solutions were put on a concave glass microscope slide and covered with a glass coverslip [33].

#### 2.10.5. Microstructure

The microstructures of Pickering emulsions were assessed using an optical microscope (LABOMED, LX400, Los Angeles, CA, USA) equipped with a digital camera (9 Megapixel, TSVIEW 6.2, Fouzhou, China). The stock emulsions were diluted 2 times in the appropriate aqueous phase to assist droplet visibility. A few drops of diluted emulsion were dripped onto the glass microscope slide and mounted with a cover slip. The images were captured at room temperature and optical magnification of ×400 [34].

#### 2.10.6. Storage Stability

The storage stability of Pickering emulsions with different concentrations of WPC–CSG NPs was measured by monitoring the volume of the separated part of samples for 2 months through visual observation at ambient temperature. Initial emulsions were poured into sealed glass tubes and stored at room temperature. The volumetric percentage of the emulsion layer to the total volume of the emulsion mixture is defined as ES, expressed as follows [35].

Emulsion stability (%) = volume of emulsion layer/total volume of emulsion mixture × 100(1)

#### 2.10.7. Thermal Stability

The thermal stability of Pickering emulsions was assessed via the emulsion creaming index. In this way, 10 mL aliquots of each emulsion were sealed in glass tubes and immersed in boiling water for 15 min. Following heat treatment, the samples were immediately cooled to room temperature. The samples were subjected to centrifugation at 6000 rpm for 10 min, and the stability was calculated according to the stable volume of the emulsion layer (Equation (1)) [36,37].

### 2.11. Statistical Analysis

The response surface methodology (RSM) and organization of the experiments with central composite design (CCD) were used to optimize the effect of the variables concentrations (CSG, WPC, and CaCl_2_ concentration) on responses (Y) including Zeta potential, particle size, and PDI. The experiment data were obtained by 22 runs. The independent variable limits were determined after pretreatment tests. Response data were analyzed using Design-Expert version 10 (Statease Inc., Minneapolis, MN, USA). The model choice was based on the significant effect of the F-test (*p* < 0.05), the correlation coefficient (R^2^), and the non-significant lack-of-fit test. Then, the optimal WPC–CSG NPs are determined based on various tests (Zeta potential, particle size, and PDI).

All factors considered in the evaluation of the appropriateness of each response model encompassed the model’s significance (*p* < 0.05), the absence of a significant lack of fit (*p* > 0.05), and the presence of an acceptable correlation coefficient. For the determination of the most suitable model, the statistical significance of the regression coefficients was conducted using Fisher’s F-test with a confidence level of 95%. The interactive effects stemming from these factors were discerned through the utilization of surface plots, derived from the chosen model. The reliability of these models was ascertained by calculating the R^2^ value for each model, where R^2^ signifies the extent of variation in the response that is elucidated by the model. Commonly, a model is suitable if R^2^ surpasses 0.9 [38].

To assess the impact of WPC–CSG NPs’ concentration, the statistical analysis was performed on the Pickering emulsion using SPSS 25 statistical software (SPSS Inc., Chicago, IL, USA). The experiment was replicated three times. The data were presented as the means ± standard deviations (SDs). Comparisons were subjected to one-way ANOVA of experiment data and evaluated with Duncan’s test at *p* < 0.05.

## 3. Results and Discussion

### 3.1. Turbidimetry of WPC–CSG Dispersion

**Figure 1:**
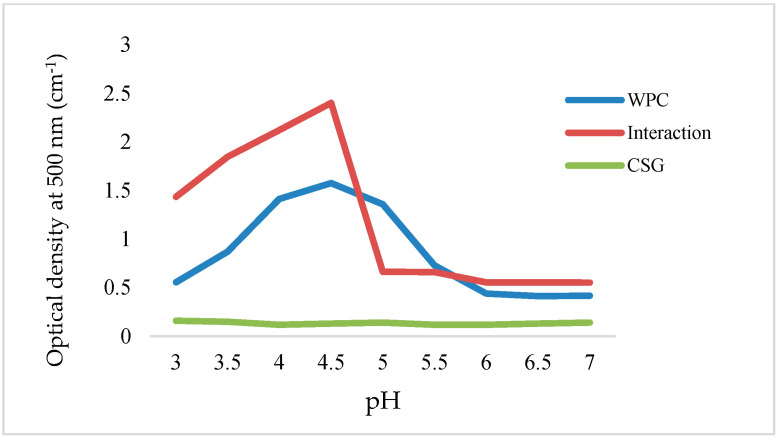
Variation in optical density with respect to pH for a solution containing 1% WPC, 0.25% CSG, and their combined mixture in a 1:1 ratio.

The pH value plays an important role in protein–polysaccharide complexes due to the electrostatic interactions. At the different pH far from *pI*, the protein ionizes, and its negative (>*pI*) or positive (<*pI*) net charge causes protein–polysaccharide soluble or insoluble complexes [18]. Figure 1 shows that the turbidity of the WPC and WPC–CSG system was affected by pH variation. The turbidity of CSG (0.25%) remained completely stable within the pH range of 3–7 (Figure 1). This stability can be attributed to both its high molecular weight and its anionic nature, resulting from a high ratio of mannose/galactose, which is characteristic of polyelectrolyte polysaccharides [39,40,41,42].

At pH values above the protein’s *pI*, they exhibit a net negative charge. Despite this, the presence of a limited number of positively charged residues allows for electrostatic interactions with negatively charged polysaccharides, leading to the formation of soluble complexes (not insoluble complexes). Unlike coacervates, these complexes are characterized by smaller particle sizes. While the Zeta potential is increased due to the net negative charge, the formation of the complex is primarily driven by the electrostatic interactions between the positively charged residues of the protein and the negatively charged groups of the polysaccharide. The soluble complex is complex resulting from insufficient pairable charged groups. In this way, the pH where the turbidity begins to increase slightly means the formation of soluble complexes and the beginning of electrostatic complexation [43].

WPC showed a U-shape solubility profile, and its absorbance raised with increasing pH toward pH 5.5. The maximum turbidity at pH 5.5 was related to *pI* due to aggregation and self-association of protein. Turbidity of WPC–CSG at pH < *pI* (pH 3–4.5) increased sharply (Figure 1), which the maximum electrostatic interaction of WPC and CSG happened between opposite charge at pH 4.5 and insoluble complexes formed [44]. On the other hand, the turbidity reduced very fast at pH < 3 which is attributed to the dissolution of the complex. Figure 1 shows the turbidity of the WPC–CSG profile reduced at pH > *pI*. This is due to the negative net charge of protein that is the same charge of polysaccharides. In other words, fewer positively charged moieties of the protein were accessible for interaction with the polysaccharide. Consequently, decreasing the pH from 7 to 6 resulted in an increase in the turbidity of WPC–CSG complexes, suggesting that the initial increase in turbidity was associated with the formation of soluble complexes. These results were in agreement with the other reports [18,45,46]. Based on the results, pH 6 was selected as the optimal pH for the production of soluble complexes of WPC–CSG nanoparticles.

### 3.2. Optimization of WPC–CSG NPs Production by RSM

The effects of WPC, CSG, and CaCl_2_ concentration on the particle size, PDI, and Zeta potential content were summarized in Table 1. In order to examine the influence of WPC, CSG, and CaCl_2_ on particle size, PDI, and Zeta potential, a range of distinct levels for WPC (1–4%), CSG (0–1%), and CaCl_2_ (1–3 mM) were investigated utilizing a CCD statistical design. The sought-after response variables encompassed the size, PDI, and Zeta potential attributes.

The desired range for each independent variable (WPC, CSG, and CaCl_2_) was selected based on a review of the literature and pre-tests that were performed. The goal of the optimization was to produce WPC–CSG NPs with the minimum particle size, the minimum PDI, and the maximum absolute value of Zeta potential [47,48,49]. Employing a second-order polynomial equation, the experimental data were fitted and responses were predicted. The equation significance was ascertained by analysis of variance (ANOVA). The acquired experimental data were suitably adjusted to a second-order polynomial equation (Equation (2)).

(2) $$Y=\beta 0+\sum \beta_{i}x_{i}+\sum \beta_{ii}x_{i}^{2}+\sum \sum \beta_{ij}x_{i}x_{j}$$

where Y is the predicted responses (Zeta potential (mV), particle size (nm), and PDI), β_0_ is the model intercept, β_i_, β_ii_, and β_ij_ are model coefficients (linear, squared, and interactive effects), whereas the independent variables are X_i_ and X_j_ (CSG, WPC, and CaCl_2_ concentration).

Each response variable underwent a comprehensive analysis, which included model summaries and lack of fit tests, across linear, quadratic, and cubic models. Table 2 and Table 3 show *p*-values, lack of fit, and correlation coefficients (*R*^2^) for the models. Also, the ANOVA tables (Table 2 and Table 3) depict an examination of the primary influences of the variables and their interconnectedness within each model. Hence, it appears that the utilization of a linear model holds promise for predicting responses and optimizing independent variables.

The predicted and experimental data in each column were compared using a *t*-test. If the difference was not statistically significant, the predicted data were accepted.

#### 3.2.1. Particle Size

The linear models generated for the relationship between the three independent variables CSG (X_1_), WPC (X_2_), and CaCl_2_ (X_3_) contents and particles’ size are shown in Equation (3):

Particle size = +112.05 + 8.814X_1_ + 38.802X_2_ + 31.483 X_3_ (3)

where X_1_ is gum concentration (CSG), X_2_ is whey protein concentration (WPC), and X_3_ is the concentration of CaCl_2_ (mM), respectively.

**Figure 2:**
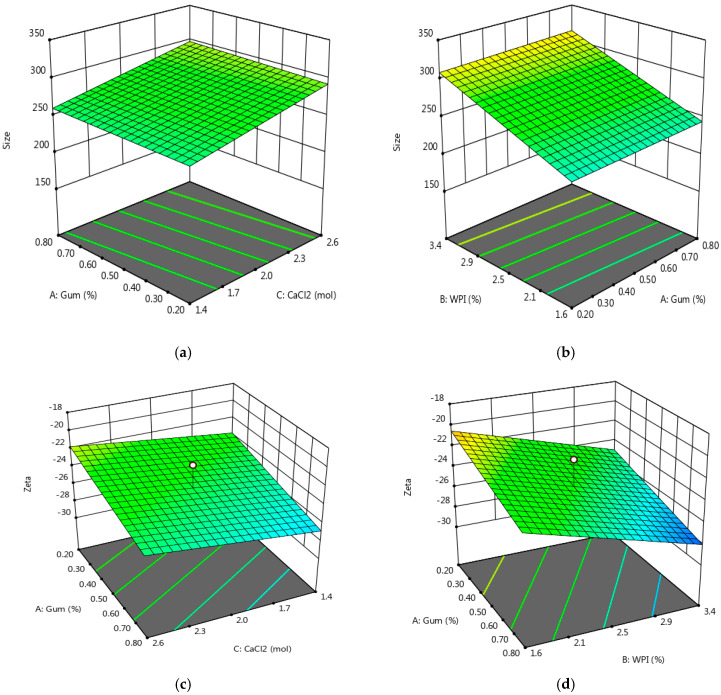
Surface plots (3D) for particle size (in (**a**), WPC = 2.5% and in (**b**), CaCl_2_ = 2 mM) and Zeta potential value of WPC–CSG NPs (in (**c**), WPC = 2.5% and in (**d**), CaCl_2_ = 2 mM).

The results of response surface analysis for the effect of CSG and WPC on particle size are shown in Figure 2a,b. Increasing the CSG from 0.2 to 0.8% *w*/*w* at pH = 6, gradually decreased the particle size (*p* < 0.05). The minimum size of nanoparticles was 189.2 nm with increasing CSG up to 0.5% in the constant concentration of 2.5% (*w*/*w*) WPC and 2 mM CaCl_2_ (Figure 2a,b). Our results are in agreement with those of prior studies that they obtained optimum particle size at 0.1% of CSG [50].

In fact, in a protein–polysaccharide dispersion at pH > *pI*, polysaccharide increased protein negative identical charge and higher electrostatic repulsing between WPC chains. On the other hand, due to mild heat treatment of protein (70 °C), WPC active surface can create interaction with polysaccharides through hydrogen bonds and hydrophobic force, which increases the interaction between WPC and CSG and decreases protein aggregation [5,7,51,52]. Thus, CSG is a suitable ingredient for producing nanoparticles and decreases the growth of WPC–CSG complex size. The effect of WPC concentration on nanoparticle size is depicted in Figure 2b. Results revealed that increment of WPC to 4% (*w*/*w*) obtained maximum nanoparticle size up to 350 nm in all studied CSG concentrations. The CaCl_2_ effect on particle size mostly depends on the WPC and CSG concentration. The increment of CaCl_2_ concentration increased nanoparticle size due to the cross-link between polymer chains (Figure 2a). It also reported that more increasing positive ion of Ca^2+^ causes partial coagulation, aggregation, and flocculation, resulting in an increase in nanoparticle size [53,54]. The PDI was between 0.161 and 0.339, but the difference was not significant between treatments (*p* < 0.05). A PDI of less than 0.5 indicates the formation of nanoparticles with a narrow size distribution [55].

**Figure 3:**
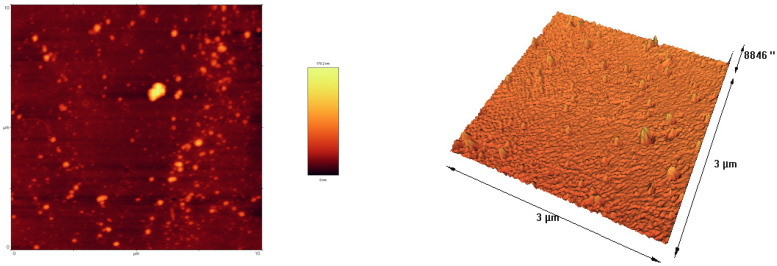
Conventional three-dimensional AFM visualizations depicting the WPC–CSG NPs dispersion at a concentration of 1.94% WPC, 0.31% CSG, and 2 mM CaCl_2_.

AFM Image showed WPC–CSG NPs displayed a consistent spherical morphology with a smooth surface, which dispersed uniformly (Figure 3).

#### 3.2.2. Zeta Potential

The linear models generated for the relationship between the three independent variables CSG (X_1_), WPC (X_2_), and CaCl_2_ (X_3_) concentration and Zeta potential are shown in Equation (4):

Zeta potential = −18.873 − 5.187X_1_ − 2.462X_2_ + 1.6713X_3_ (4)

where X_1_ is gum concentration (CSG), X_2_ is whey protein concentration (WPC), and X_3_ is the concentration of CaCl_2_ (mM), respectively.

The results showed the absolute value of Zeta potential increased with an increase in WPC and CSG concentration and decreased with an increase in CaCl_2_ concentration (Table 1). This observation can be clarified by the fact that whey protein at pH greater than its *pI* acquires a negative charge due to the protonation of its COOH groups [56]. Also, CSG nature is anionic, and less electrostatic interaction with WPC occurred. In other words, WPC had lower positive groups which entanglement lower numbers of carboxyl groups of CSG, consequently increasing the Zeta potential absolute value. The surface plots (Figure 2c,d) show that all the selected independent variables were statistically significant. Prior research has demonstrated that CaCl_2_ salt had a significant impact on hydrophobic and electrostatic interactions [57].

### 3.3. Optimization of WPC–CSG NPs by RSM

A numerical optimization was performed to estimate the optimal operating conditions for the production of WPC–CSG NPs. The optimized system aimed to maximize the absolute value of Zeta potential and minimize the particle size as well as PDI. Table 4 gives WPC–CSG NPs produced at optimum conditions (CSG 0.31%, WPC 1.75%, and CaCl_2_ 1.69 mM). The correlation between predicted and experimentally observed values of Zeta potential, particle size, and PDI was determined after the preparation of nanoparticles at optimized conditions. In the case of PDI, we could not fit a significant model at all. Therefore, we did not consider this response in optimization. The optimum conditions predicted by the Liner model were at Zeta potential −23 mV, particle size 223.04 nm, and the PDI 0.23, which were verified by experimental results obtained at optimal extraction conditions (Zeta potential −21.53 mV, particle size 220.48 nm, and the PDI 0.23). The desirability of the model was 0.89.

### 3.4. Contact Angle of WPC–CSG NPs

Based on our previous results, WPC–CSG NPs were successfully prepared with an average particle size of 223.04 nm and a Zeta potential of −43 mV. The wettability of the nanoparticles is an important factor for identifying the Pickering emulsions type and their stability [58,59]. The partial wettability of solid particles allows them to interact with both the oil and water phases in a Pickering emulsion, leading to the formation of bridges between droplets and enhancing the emulsion’s stability. Although whey protein is known for its surface activity and rapid adsorption kinetics, its inherent hydrophobicity can actually decrease its adherence to oil–water [60]. The interaction between proteins and ionic polysaccharides can influence the functional properties of proteins, such as solubility and emulsifying ability [61]. In this way, the CSG as an anionic polysaccharide by interacting with the opposite charges of protein enhances the wettability of nanoparticles, allowing them to spread more effectively across the oil–water interface [26,42,62].

The contact angles for the samples at concentrations of 0.5, 0.7, and 1% of WPC–CSG NPs were 41.44°, 58.54°, and 61.13°, respectively. These results suggest a higher exposure of hydrophilic groups on the surface [63]. Although the contact angle of the Pickering emulsion increased as the WPC–CSG NPs’ concentration rose from 0.5% to 1%, all samples exhibited contact angles below 90°. Based on the Bancroft rule, hydrophilic particles with a contact angle < 90° are suitable for stabilizing O/W emulsions, and hydrophobic particles with a contact angle > 90° are more appropriate for stabilizing water in oil emulsion [64]. Several studies have demonstrated that particles exhibiting contact angles around 60° can effectively generate relatively stable O/W Pickering emulsions [65,66].

Ataeian et al. (2023) conducted a study to examine the impact of the hydrophobic modification on the wettability of cellulose nanocrystals within an O/W Pickering emulsion, employing contact angle measurements. The findings revealed nanoparticles with a water contact angle of 56° resulted in Pickering emulsion with smaller droplet sizes [67].

### 3.5. Interfacial Tension

**Figure 4:**
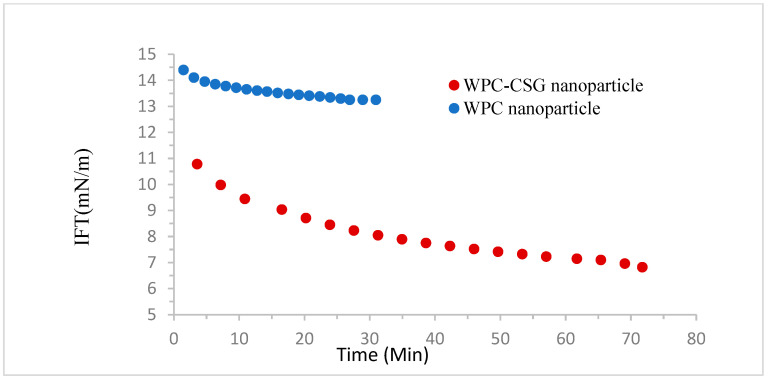
Interfacial tension of WPC NPs (1.75% *w*/*v*) and WPC–CSG NPs (1.75% *w/v* WPC: 0.31% *w/v* CSG).

The interfacial tension of optimized WPC–CSG NPs (0.31% *w/v* CSG, 1.75% *w/v* WPC, and 1.69 mM CaCl_2_) was measured and compared with WPC (1.75% *w*/*v*) in nanoparticle form. The result showed that the interfacial tension value of WPC NPs was higher than WPC–CSG NPs (Figure 4). The rapid decrease in interfacial tension upon nanoparticle addition signifies the formation of an interfacial layer. Subsequently, the interfacial tension reduction in both WPC–CSG and WPC NPs gradually slowed down with time. This observation indicates that the adsorption of nanoparticles initially proceeded rapidly, as there were numerous unoccupied adsorption sites at the oil–water interface. However, the adsorption rate subsequently slowed down due to the filling of these sites by the nanoparticles [68,69].

In the work of Yao et al. (2021), it was also noted that the whey protein isolate–Arabic gum nanoparticles displayed a lower interfacial tension in contrast to the whey protein isolate [70].

It is accepted that the initial interface tension is determined by the diffusion of solid particles from the continuous phase to the interface, which can be affected by the size, shape, surface charge, and hydrophobicity of the particle [71]. According to our previous work, the WPC–CSG NPs, produced in different concentrations, had more absolute Zeta potential value and smaller particle size than WPC NPs.

Song et al. (2022) found that the interfacial tension of soybean protein isolate–maltose nanoparticles decreased from 46.62 to 44.34 mN/m as the concentration of nanoparticles complex increased from 15 mg/mL to 35 mg/mL. The authors also highlighted the interaction between soybean protein isolate and maltose, which resulted in increased protein expansion and enhanced protein flexibility. This augmentation in contact probability prompted greater protein unfolding and improved protein flexibility, ultimately contributing to a reduction in the interfacial tension within the oil–water interface. This decrease in interfacial tension has the potential to accelerate the formation of bridges between droplets, thereby abbreviating the duration of droplet contact. Consequently, a shorter contact duration among droplets translates to heightened stability of the emulsion [72].

### 3.6. Pickering Emulsion Properties

#### 3.6.1. Zeta Potential and Droplet Size

Pickering emulsions were produced with different concentrations of WPC–CSG NPs and the average size, Zeta potential, and PDI were determined. The absolute value of Zeta potential significantly increased (*p* < 0.05) with increasing of nanoparticle concentration from 0.5 to 1% (*w*/*v*) (Table 5). The high value of Zeta potential indicated the nanoparticle adsorbed on the droplet surface, and the presence of more WPC–CSG NPs increased the negative charge of the droplet interface and subsequently increased Zeta potential [73]. In this study, the WPC–CSG soluble complex was formed at pH 6 (above the whey protein isoelectric point) being in agreement with others’ works [74,75]. At this pH, the nanoparticles had a negative charge of −43 mV. Therefore, the Zeta potential of the Pickering emulsions became negative.

Kim et al. (2023) observed that the addition of a small anionic surfactant to whey protein complexes enhanced the negative charge of Pickering emulsions. The Zeta potential of the WPC–CSG Pickering emulsion system was greater than the whey protein Pickering emulsion, which resulted in improving emulsion stability across a broader pH range (pH 3–7) [76].

The droplet size of the Pickering emulsions significantly (*p* < 0.05) decreased for higher WPC–CSG concentrations (Table 5). A nanoparticle concentration of 0.5, 0.7, and 1% (*w*/*v*), resulted in an average droplet size of 3 ± 0.57 μm to 2 ± 0.50 μm and 0.91 ± 0.60 nm, respectively. This fact can be explained by the formation of a denser layer of protein–polysaccharide nanoparticles between adjacent droplets, protecting oil droplet attachment and decreasing droplet size [77,78]. When additional nanoparticles are present and are enough to adsorb onto the oil droplets, more nanoparticles participate in the formation of the interfacial layer, thus resulting in more droplets with smaller sizes. In general, the smaller the droplet size, there is an increased role in improving Pickering emulsion stability against flocculation, coalescence, and Ostwald ripening [79,80].

As reported, smaller particles need lower energy to adsorb on the O/W interface [81]. In other words, smaller particles have a longer adsorption time at the oil–water interface, resulting in a decrease in the diameter of the emulsion droplets [82]. Therefore, an increase in WPC–CSG (0.5 to 0.7 and 1% *w*/*w*), due to their nanometer size (223.04 nm), resulted in the formation of small droplets.

The PDI of all samples was less than 0.5, and there was no significant difference between samples (*p* > 0.05). It seems that uniform dispersion resulted from the characteristics of WPC–CSG NPs [83].

#### 3.6.2. Morphology of Pickering Emulsion

**Figure 5:**
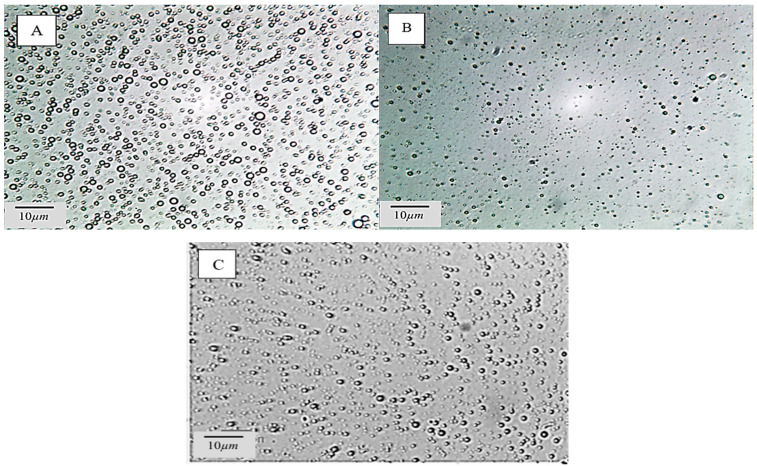
Optical microscopic images of Pickering emulsions stabilized by different concentrations of WPC–CSG NPs (0.5% (**A**), 0.7% (**B**), and 1% (**C**) *w*/*v*).

The morphology of Pickering emulsions was captured by optical microscopy (Figure 5). The droplets of samples (stabilized by 0.5, 0.7, and 1% WPC–CSG) had a spherical appearance with obvious boundaries. When the concentration of WPC–CSG was 0.7 and 1% (*w*/*v*), the particles became smaller and more uniform than 0.5% (*w*/*v*), which might be due to sufficient nanoparticles being able to cover the surface of oil droplet and more oil droplets by smaller size. Nevertheless, the images showed a mono-modal droplet size distribution without aggregation and coalescence phenomenon so the nanoparticle can be good emulsifiers [34]. Also, optical microscopy results were consistent with the confocal microscopy that revealed that an increase in the WPC–CSG NPs’ concentration completely inhibits the aggregation of emulsion droplets.

These images are consistent with the low PDI values obtained from DLS measurements, which suggest a narrow and more uniform droplet distribution of Pickering emulsion with higher concentrations of WPC–CSG NPs. Therefore, the results suggest that the WPC–CSG NPs played a significant role in enhancing the uniformity of droplet distribution and the microstructure of the Pickering emulsions.

#### 3.6.3. CLSM Image

**Figure 6:**
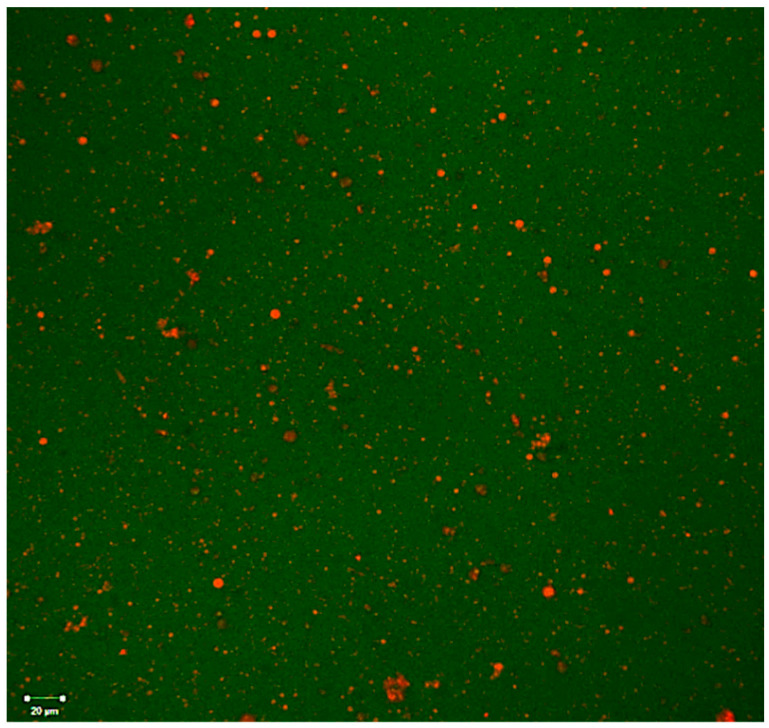
CLSM images of Pickering emulsion stabilized by 1% (*w/v*) of WPC–CSG NPs.

The interfacial properties of the Pickering emulsions were observed by CLSM (Figure 6). The CLSM images of Pickering emulsion stabilized by 1% WPC–CSG NPs show the nanoparticle adsorbed on the surface of dispersed small oil droplets to form an interfacial film. This structure could improve the stability of Pickering emulsions [84]. The images show that the Pickering emulsions were the oil-in-water type. Also, droplets’ aggregation could not be observed in the Pickering emulsion with 1% WPC–CSG. This can be explained by the fact that the interfacial film composed of 1% WPC–CSG NPs was sufficient to cover the surface of oil droplets, which caused the droplet resistance to coalescence and Ostwald ripening [85].

#### 3.6.4. Rheology

**Figure 7:**
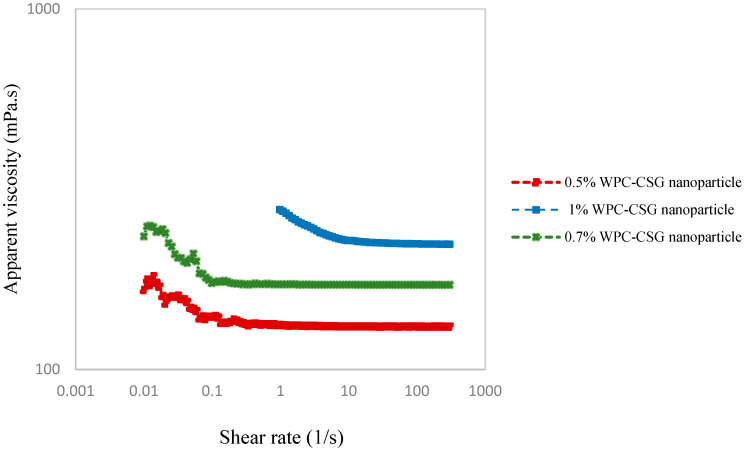
Flow behavior for Pickering emulsions stabilized by different concentrations of WPC–CSG NPs (0.5, 0.7, and 1%, *w*/*v*).

The rheological properties of Pickering emulsions at 0.5, 0.7, and 1% WPC–CSG NPs’ concentrations are presented in Figure 7. The viscosity decreased by increasing in shear rate, which showed the shear thinning behavior of all Pickering emulsions. The apparent viscosity of Pickering emulsion stabilized by 0.5, 0.7, and 1% WPC–CSG NPs was 131, 171, and 221 m·Pa·s in shear rate 300 s^−1^, respectively. The viscosity of the Pickering emulsions is controlled by the viscosity of the continuous phase and densely packed interfacial layer. In other words, the excessive nanoparticles that did not adsorb to the interface created gel-like structures in the continuous phase and further increased the viscosity. The rheology of the continuous phase plays an important role in the stability of the emulsion; it prevents the interaction of small droplets and decreases the mobility of oil droplets [86,87,88]. The registered higher viscosity of the Pickering emulsion stabilized by 1% WPC–CSG NPs in comparison to 0.5% WPC–CSG NPs may be related to the effect of this particle in the continuous phase.

Boostani et al. (2020) reported a higher apparent viscosity of emulsion, which could be due to interactions between smaller droplets with larger specific surface area and flocculation might lead to an increase in the resistance to flow [89].

In other words, a higher viscosity of a Pickering emulsion can be attributed to a combination of factors, including the viscosity of the continuous phase, the droplet size distribution, the interfacial properties, and the interactions between particles or droplets (which can be influenced by flocculation). Among the various factors influencing emulsion viscosity, flocculation is a notable contributor, impacting the flow characteristics of droplets [90]. Our findings suggest that the low viscosity of the Pickering emulsion is a consequence of the lack of flocculation. The dispersed phase, remaining in a dispersed state without forming aggregates, contributes to a lower overall viscosity.

As shown in Figure 7, Pickering emulsions stabilized with different concentrations (0.5, 0.7, and 1%, *w*/*v*) and showed a shear-thinning (pseudoplastic) behavior as the shear rate increased from 0 to 100 s^−1^. This shear-thinning behavior exposed the network structure disrupted with an increase in shear rate [86].

#### 3.6.5. Storage and Temperature Stability

**Figure 8:**
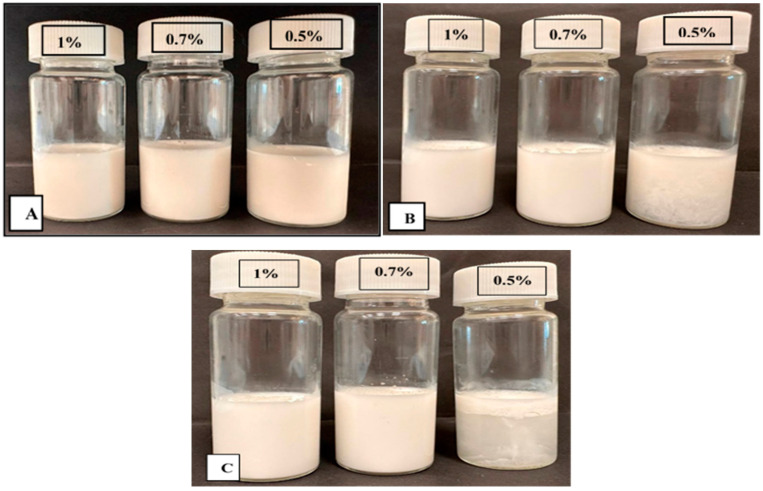
Visual observation of Pickering emulsions stabilized by different WPC–CSG NPs’ concentrations (0.5% *w*/*v*, 0.7% *w/v*, and 1% *w*/*v*) during storage (10 days (**A**), 30 days (**B**), and (**C**) 60 days at 25 °C).

The stability of Pickering emulsions was investigated during storage through visual observation and demonstrated in Table 6 and Figure 8. The result showed the Pickering emulsion stabilized with 1% WPC–CSG concentration and displayed exceptional stability, exhibiting no observable separated layer even after 60 days of storage (Table 6). In contrast, a higher separated part value was observed (23.33 ± 3.33%) in the Pickering emulsion stabilized with 0.5% WPC–CSG NPs during 60 days. This phenomenon was confirmed by the results of the droplet size and Zeta potential of emulsions (Table 6). It is accepted that the higher absolute Zeta potential value can increase the electrostatic repulsion between droplets and increase the stability of emulsions against creaming [81,91]. The emulsions formed using polysaccharides increase the viscosity of the aqueous phase, which hinders droplet mobility and improves creaming stability [91,92,93]. The viscosity measurements showed that the Pickering emulsion stabilized by a higher concentration of WPC–CSG NPs (1% *w*/*v*) had higher viscosity, and maybe there were also sufficient particles to cover the oil droplets completely.

As shown in Table 6, the different concentrations of WPC–CSG NPs-stabilized emulsions exhibited significant differences after heating (*p* < 0.05). This phenomenon of thermal stability of proteins–polysaccharides can be attributed to the fact that the polysaccharide molecules provide a sufficient steric hindrance to increasing Pickering emulsion creaming stability under heating conditions [94].

As substantiated by numerous studies, there is a well-established correlation between a higher concentration of nanoparticle complexes and the augmentation of steric resistance, coupled with enhanced electrostatic repulsion among oil droplets. This synergy gives rise to an elevated viscosity within the continuous phase, collectively fostering enhanced stability for Pickering emulsions [86,88,93,95,96,97]. Furthermore, at higher concentrations of solid particles, the adsorption layer formed at the oil–water interface becomes thicker. As a result, this reduction in oil droplet coalescence leads to an increase in the stability of the emulsion [97]. The previous studies confirmed that the use of corn oil as a carrier oil for beta-carotene can be effective in maintaining its stability. Emulsions formulated with long-chain triglycerides (LCT), such as corn oil, exhibit enhanced oxidative stability compared to those made with medium- and short-chain (MCT and SCT) triglycerides [98]. Also, emulsions formed from corn oil (as an LCT oil) had smaller particle sizes, which increased the deformation resistance of the emulsions and exhibited good stability during simulated in vitro digestion [99]. Therefore, using corn oil helped to improve the emulsion system stability. The rheological properties of the interfacial layer exert a significant influence on emulsion stability due to their impact on environmental tension; a strong interfacial layer can effectively mitigate coalescence. Polysaccharides possess the ability to engender a substantial and resilient interfacial layer, impeding droplet aggregation, even in the presence of thermal stress. This attribute contributes to the enhanced thermal stability of the Pickering emulsion [100]. Yekta et al. (2023) reported that the formation of strong interactions between soy proteins and chitosan improved the thermal stability of the proteins [61].

## 4. Conclusions

Response surface methodology was used to optimize the fabrication of WPC–CSG NPs based on Zeta potential and particle size. The WPC–CSG NPs exhibited distinct characteristics, including a higher absolute value of Zeta potential and a lower oil–water interfacial tension, both of which contribute to the formation of oil-in-water Pickering emulsions. The balanced wettability resulting from the interaction of CSG with WPC, along with the surface activity potential and spherical morphology of WPC–CSG NPs, empowers them to form an adsorption layer at the oil–water interface. This, in turn, leads to a significant reduction in the size of oil droplets within the emulsion when higher NPs’ concentrations are used. Furthermore, the soluble protein–polysaccharide complex benefits from an abundance of negatively charged groups on the surface of WPC–CSG, fostering robust electrostatic repulsion that effectively mitigates the aggregation of oil droplets. The behavior exhibited by Pickering emulsions stabilized using WPC–CSG is characterized by its dependence on concentration levels and can be systematically classified into three distinct modes: the agglomeration and flocculation of droplets at lower concentrations (0.5%); the formation of non-uniformly sized droplets at intermediate concentrations (0.7%); and the dispersion of droplets within interconnected continuous networks at higher concentrations (1%). Furthermore, the stabilized Pickering emulsion, featuring a high concentration of WPC–CSG NPs, exhibited exceptional storage stability. In conclusion, the utilization of high concentrations of WPC–CSG NPs represents a breakthrough in enhancing the stability of food-grade Pickering emulsions.

## Figures and Tables

**Table 1:** Composition of various runs of independent variables and the responses of the dependent variables utilized in the central composite design (CCD) of the particle size, Zeta potential, and PDI.

	Independent Variables			Response		
	CSG (%)	WPC (%)	CaCl_2_ (mM)	Zeta Potential (mV)	Particle Size (nm)	PDI
1	0.50	1.0	2.0	−20	189.2	0.218
2	0.25	1.0	2.0	−18.64	242.1	0.339
3	0.80	3.4	2.6	−27.22	340.2	0.258
4	0.50	2.5	3.0	−21.92	300.2	0.241
5	0.25	1.0	2.0	−18.69	240.4	0.192
6	0.25	1.0	2.0	−18.75	225.3	0.215
7	0.20	3.4	1.4	−26.12	290	0.201
8	0.25	1.0	2.0	−18.74	210.9	0.215
9	0.25	1.0	2.0	−18.76	222.1	0.215
10	0.50	2.5	2.0	−24.41	268.2	0.214
11	0.50	2.5	2.0	−21.77	270	0.225
12	1.00	2.5	2.0	−26.54	299.1	0.220
13	0.20	1.6	2.6	−21.64	250.2	0.161
14	0.50	2.5	1.0	−25.54	240.7	0.220
15	0.50	4.0	2.0	−27.22	350	0.271
16	0.80	1.6	2.6	−23.04	255.2	0.161
17	0.20	3.4	2.6	−24.85	322.2	0.191
18	0.80	1.6	1.4	−26.24	220.2	0.205
19	0.50	2.5	2.0	−25.94	275.6	0.206
20	0.80	3.4	1.4	−28.95	290.2	0.203
21	0.20	1.6	1.4	−23	210.5	0.179
22	0.00	2.5	2.0	−21.22	267	0.184

**Table 2:** Result of ANOVA and fit statistics for the particle size.

Source	Sum of Squares	DF	Mean Square	F Value	*p*-Value
Model	35,561.95	3	11,853.98	62.96	0.0001
Gum	105.25	1	105.25	0.56	0.4643
Whey protein	27,457.63	1	27,457.63	145.84	0.0001
CaCl_2_	4837.14	1	4837.14	25.69	0.0001
Residual	3388.88	18	188.27		
Lack of fit	2672.14	12	222.68	1.86	0.2289
Pure error	716.74	6	119.46		
Total	38,950.83	21			
Fit statistics					
R^2^	0.9130		Adjusted R^2^	0.8985	
C.V. %	5.22		Predicted R^2^	0.8762	

**Table 3:** Result of ANOVA and fit statistics for the Zeta potential.

Source	Sum of Squares	DF	Mean Square	F Value	*p*-Value
Model	209.21	3	69.74	54.37	<0.0001
Gum	36.46	1	36.46	28.43	<0.0001
Whey protein	110.53	1	110.53	86.17	<0.0001
CaCl_2_	13.63	1	13.63	10.63	0.0043
Residual	23.09	18	1.28		
Lack of fit	14.18	12	1.18	0.80	0.6545
Pure error	8.91	6	1.48		
Total	232.30	21			
Fit statistics					
R^2^	0.9006		Adjusted R^2^	0.8840	
C.V. %	4.89		Predicted R^2^	0.8630	

**Table 4:** Optimized conditions of the production of WPC–CSG NPs (CSG, WPC, and CaCl_2_) and response range (dependent variable: Zeta potential, particle size, and PDI).

Name	Goal	Lower Limit	Upper Limit	Lower Weight	Upper Weight	Importance
A: CSG	is in range	0.202698	0.797302	1	1	3
B: WPC	is in range	1.60809	3.39191	1	1	3
C: CaCl_2_	is in range	1.4054	2.5946	1	1	3
Zeta	maximize	−28.95	−18.64	1	1	3
size	minimize	189.2	350	1	1	3
PDI	none	0.161	0.342	1	1	3

**Table 5:** Values of Zeta potential, average droplet size, and PDI of Pickering emulsions with different contents of WPC–CSG.

WPC–CSG Concentration (% *w*/*v*)	Zeta Potential (mV)	Average Droplet Size (μm)	PDI
0.5	−38.66 ± 0.66 ^b^	3.00 ± 0.57 ^b^	0.42 ± 0.03 ^a^
0.7	−44.00 ± 1.00 ^a^	2.00 ± 0.50 ^ab^	0.33 ± 0.02 ^b^
1	−44.33 ± 0.67 ^a^	0.91 ± 0.60 ^a^	0.30 ± 0.02 ^b^

Values are means of three replicates ± SD. ^a,b^ Different letters in the same column indicate significant differences at *p* < 0.05.

**Table 6:** Stability of Pickering emulsions with different concentrations of WPC–CSG NPs’ concentration (0.5% *w*/*v*, 0.7% *w/v*, and 1% *w*/*v*) during storage and under temperature stability test.

WPC–CSG NPs Concentration (% *w*/*v*)	Storage Stability (10 Days) (%)	Storage Stability (30 Days) (%)	Storage Stability (60 Days) (%)	Thermal Stability (%)
0.5	100.00 ± 0.00 ^aA^	65.21 ± 2.91 ^bB^	23.33 ± 3.33 ^cC^	51.25 ± 1.20 ^c^
0.7	100.00 ± 0.00 ^aA^	100.00 ± 0.00 ^aA^	96.68 ± 2.87 ^aA^	72.50 ± 2.08 ^b^
1	100.00 ± 0.00 ^aA^	100.00 ± 0.00 ^aA^	100.00 ± 0.00 ^aA^	83.25 ± 2.75 ^a^

Different letters indicate significant differences between emulsions (small letters) and during storage (capital letters) at *p* < 0.05.

## Data Availability

The original contributions presented in the study are included in the article, further inquiries can be directed to the corresponding author.
